# Evaluating the Association between Diabetes, Cognitive Decline and Dementia

**DOI:** 10.3390/ijerph120708281

**Published:** 2015-07-17

**Authors:** Omorogieva Ojo, Joanne Brooke

**Affiliations:** 1 Faculty of Education and Health, University of Greenwich, Avery Hill Campus, Avery Hill Road, London SE9 2UG, UK; 2 College of Nursing, Midwifery and Healthcare, University of West London, Paragon House, Boston Manor Road, Middlesex TW8 9GA, UK; E-Mail: joanne.brooke@uwl.ac.uk

**Keywords:** diabetes, dementia, Alzheimer’s disease, cognitive decline, lifestyle interventions, self-management of diabetes, diabetes complications

## Abstract

The aim of this article is to review the association between diabetes mellitus, cognitive decline and dementia, including the effects of cognitive decline and dementia on self management of diabetes. This is a literature review of primary research articles. A number of contemporary research articles that met the inclusion criteria were selected for this review paper. These articles were selected using a number of search strategies and electronic databases, such as EBSCOhost Research and SwetsWise databases. The duration of diabetes, glycated haemoglobin levels and glycaemic fluctuations were associated with cognitive decline and dementia. Similarly, hypoglycaemia was significantly related to increased risk of developing cognitive decline and dementia. Furthermore, cognitive decline and dementia were associated with poorer diabetes management. There is evidence of the association between diabetes, cognitive decline and dementia including the shared pathogenesis between diabetes and Alzheimer’s disease. In addition, the self management of diabetes is affected by dementia and cognitive decline. It could be suggested that the association between diabetes and dementia is bidirectional with the potential to proceed to a vicious cycle. Further studies are needed in order to fully establish the relationship between diabetes, cognitive decline and dementia. Patients who have diabetes and dementia could benefit from structured education strategies, which should involve empowerment programmes and lifestyle changes. The detection of cognitive decline should highlight the need for education strategies.

## 1. Introduction

Diabetes Mellitus (DM) is a metabolic disorder that results from beta cell dysfunction and/or the failure of insulin to exert its biological influence at the level of the muscle or the liver, thus leading to chronic hyperglycaemia [1]. Diabetes often involves disturbances of carbohydrate, fat and protein metabolism with severe ramifications resulting in long term complications. These complications impact on the physiological systems and organs of the body and may lead to cerebrovascular dysfunction and dementia. The prevalence of Diabetes is on the increase in both the UK and worldwide partly due to advances in technology which enable more effective and better diagnosis, lifestyle choices and an ageing population [1,2].

On the other hand, dementia is the most severe pathological form of brain ageing [3]. All dementias, including Alzheimer’s disease, Vascular dementia, Frontal—temporal and Lew body dementia, involve a progressive decline in multiple areas of higher cortical function, including memory, reasoning, communication, and ability to carry out activities of daily living [4,5]. Alongside this decline, individuals may experience behavioural and psychological symptoms, such as depression, aggression, psychosis, and wandering [6]. The prevalence of dementia is also on the increase both in the UK and worldwide, again, partly due to the ageing population [7].

### 1.1. Prevalence of Diabetes and Dementia

The worldwide prevalence of diabetes is estimated to be 285 million adults, with this number expected to rise to 439 million in 2030 [8]. However, type 2 diabetes may be present in up to 80% of individuals with dementia who are aged 65 years or older [9]. Type 2 diabetes may increase the risk of developing Alzheimer’s disease from the “pre-dementia” condition of mild cognitive impairment [9,10].

Globally, 35.6 million people are living with dementia, with this number projected to double every 20 years [8,9]. In the UK 700,000 people are estimated to have dementia and this figure is expected to rise to over 1.7 million by 2051 [4], with one in 14 people over the age of 65 years and one in six over the age of 80 years living with dementia [3].

Risk factors for dementia include age and genetic predisposition [11]. In addition, the association between glucose, insulin and lipid metabolism and the underlying neurobiological changes observed in patients with Alzheimer’s disease are being established (Box 1). Longitudinal prospective studies support the link between type 2 diabetes and hyperinsulinaemia with the development of Alzheimer’s disease, however not all research confirms this relationship [10].

In recent years, the role of diabetes in the development of cognitive decline and dementia has been the subject of research studies and discussion [10,12]. Box 1 shows the relationships between key factors including hyperglycaemia, hyperinsulinaemia and comorbid vascular conditions, which are associated with diabetes and other metabolic pathways that link diabetes to Alzheimer’s disease [10]. Interestingly, concerns remain with respect to the possible impact of cognitive impairment and memory loss on the self management of diabetes. Self-management of diabetes is the ability of the individual with diabetes to make choices and decisions about their lives and how they manage their condition [13]. This is aimed at improving the quality of their lives and reducing the risk of developing complications. While self management is the process of developing appropriate skills by individuals with diabetes to manage their condition, self care relates to the daily activities carried out to control the disease [13]. Some of the essential components of self management related to diabetes care include managing acute complications such as hypoglycaemia and hyperglycaemia, managing associations between food, physical activity and medications, and self monitoring of blood glucose and blood pressure [14]. In addition, persons with diabetes need support from health care professionals, peers, family, friends and carers, and access to structured education, personalised care plan, and emotional and psychological support in order to promote self management [13].

Box 1. **Key elements and metabolic pathways linking Type 2 diabetes and Alzheimer’s disease.**

Hyperglycaemia
Hyperinsulinaemia
Comorbid Vascular Conditions (Hypertension, Obesity)
Dyslipidaemia (High triglycerides, Low High density lipoprotein cholesterol, Dense Low density lipoprotein particles)
Cerebrovascular dysregulation (Endothelial dysfunction, Microinfarcts, White matter changes) Amyloid – β metabolism (Amyloid – β generation, Reduced Amyloid – β clearance)
Inflammation
Source: [10]

Therefore, a review of the possible impact of diabetes on cognitive impairment and the consequence of this on the management of diabetes and associated complications becomes pertinent. The effect of diabetes on cognitive impairment may result from the physiological changes associated with diabetes and the role of poor glucose control which may lead to chronic hyperglycaemia and hypoglycaemia [14,15,16,17]. In addition, the reverse impact of dementia on diabetes appears to complete this cycle of relationships which could be defined as a bidirectional model. Within this article, the terms cognitive impairment and cognitive decline are used interchangeably.

### 1.2. Aim

This article is a literature review of published research studies. The aim is to evaluate the bidirectional association between diabetes, cognitive decline and dementia.

## 2. Methods

A review was carried out based on published guidelines [18]. The literature search involved relevant articles relating to diabetes, cognitive decline, dementia, and self- management of diabetes. An initial general scoping of the databases on the risk of developing dementia from diabetes found the Rotterdam study [19] and some systematic reviews [20,21]. Since these publications, a number of other primary research studies have been conducted.

The following data bases which included EBSCO Host/Health Sciences Research databases (encompassing Academic search premier, Medline, Psychology and Behavioural sciences collection, PSYCINFO, SPORTDISCUSS and Cumulative Index to Nursing and Allied Health Literature (CINAHL) Plus) and SwetsWise were searched. In addition, reference lists of identified articles were manually searched for relevant studies.

### 2.1. Inclusion and Exclusion Criteria

Primary research articles which are usually based on original and/or new data, published between 2004 and 2014 and written in English language were the only studies included in this review. Although Biessels *et al*. [20] published a systematic review in 2006, the year of publication for articles included in the current review commenced in 2004 because relevant studies such as the article published by Beeri *et al*. [22] were omitted in that systematic review.

In particular, while examining the associations between diabetes, cognitive impairment and dementia only longitudinal prospective studies were included, and cross sectional studies were selected to assess the possible effect of cognitive dysfunction on management of diabetes. Although longitudinal prospective studies may attempt to establish the link between diabetes and dementia, evidence of the role of dementia and cognitive impairment on diabetes are limited in this study design. Therefore, another type of research design in the form of cross sectional studies which provide this evidence were included in the review. Studies which did not meet the above inclusion criteria were excluded from the study. The outcome measures of interest were cognitive impairment, dementia, self-management of diabetes, hyperglycaemia, hypoglycaemia and glycated haemoglobin.

### 2.2. Data Analysis

Based on the key terms used for the search, 5538 articles were found initially. Of these articles, eleven articles which met the requirements for selection were included in the review (Table 1).

## 3. Results

### 3.1. Association between Diabetes, Cognitive Impairment and Dementia

Eight [8,22,23,24,25,26,27,28] of the eleven studies included, investigated the risk of cognitive decline and dementia in patients with diabetes. Similar trends were observed in six of the eight studies with the main findings showing that diabetes, including its duration, glycated haemoglobin (HbA1c) levels and glycaemic fluctuations were associated with cognitive decline and dementia [8,22,23,24,25,26] although one of these studies [23] showed that cognitive decline may be less evident after age 85 years. The biochemical measures of diabetes are mainly defined by the levels of blood glucose (Fasting, Postprandial and glycated haemoglobin) [1]. Two of the studies that examined the relationship between hypoglycaemia and the risk of developing cognitive impairment and dementia found that hypoglycaemia was significantly associated with increased risk of cognitive decline and dementia [27,28].

**Table 1:** Summary of Studies Reviewed.

Citation	Country	Type of Study	Sample size	Aims/Objectives	Outcomes
Sanz *et al*., 2012 [8]	France	Prospective multicentre cohort study (Longitudinal)	608	To determine whether diabetes mellitus influences functional status in patients with Alzheimer’s disease.	At baseline, the presence of diabetes significantly increases the risk of functional disability in patients with Alzheimer’s disease.
Beeri *et al.*, 2004 [22]	Israel	Longitudinal study	1892	To examine the association between diabetes in midlife and dementia more than three decades later.	There was evidence that diabetes was a risk factor for dementia.
van den Berg *et al*., 2006 [23]	The Netherlands	Prospective and longitudinal	599	To examine the impact of diabetes mellitus on cognitive decline over time in the oldest of the old.	Very old patients with diabetes have lower cognitive function compared with patients without diabetes at age 85 years, but they do not decline faster at age 85 years to 90years.
Umegaki *et al*., 2012 [24]	Japan	Longitudinal study	63	To identify the associated factors with cognitive decline.	Higher glycaeted haemoglobim (HbA1c) had a tendency toward association with cognitive decline.
Gao *et al*., 2008 [25]	United Kingdom	Multi-centre longitudinal study	1139	To investigate the association between the level of HbA1c and mortality from all causes, including cognitive decline.	Respondents in the group HbA1c ≥7% who had not been diagnosed had a significantly higher risk of developing dementia. Biomarkers of glucose metabolism (HbA1c) are associated with dementia.
Okereke *et al*., 2008 [26]	USA	Prospective cohort study (Longitudinal)	12,233	To relate diabetes mellitus status and duration to late-life cognitive impairment and decline.	Type 2 diabetes and longer duration of diabetes are similarly related to cognitive impairment and decline
Lin and Sheu, 2013 [27]	Taiwan	Longitudinal study	15,404	To investigate the risk of dementia in patients with type 2 diabetes with or without prior hypoglycemic episodes.	Adult patients with prior hypoglycaemia had a significantly increased risk of dementia
Feinkohl *et al*., 2014 [28]	UK	Prospective study (Longitudinal)	831	To determine the association of both prevalent and incident severe hypoglycaemia with cognitive decline.	Severe hypoglycaemia was associated with significant decline in cognitive function. Lower cognitive ability at baseline was associated with two fold higher incidence of severe hypoglycaemia over 4 years
Feil *et al*., 2011 [29]	USA	Cross sectional database analysis	497,900	To examine the relationship between management of diabetes and hypoglycemia in older adults with and without dementia and cognitive impairment	Dementia and cognitive impairment were independently associated with greater risk of hypoglycemia
Feil *et al*., 2012 [30]	USA	Cross sectional observational analysis	1398	To examine the relationship between cognitive impairment and diabetes self management	Cognitive impairment is associated with worse self care.
Grober *et al*., 2011 [31]	USA	Cross sectional study	169	To assess the relationship of glycemic control to memory impairment and executive dysfunction in older adults with diabetes	Memory impairment and executive dysfunction were associated with inadequately controlled diabetes. Cognitive dysfunction may interfere with diabetes management and inadequate diabetic control may contribute to cognitive decline

In a study by Sanz *et al*. [8], the longitudinal data confirmed that diabetes was associated with functional disability in patients diagnosed with Alzheimer’s disease for less than one year, but not in those diagnosed with Alzheimer’s disease longer than one year. On the other hand, Beeri *et al.* [22] found that diabetes in mid life could be linked to dementia more than three decades later.

The study by van den Berg *et al.* [23] showed that at 85 years of age, diabetes was associated with a lower level of cognitive functioning, while in another study [24], higher glycated haemoglobin was linked with cognitive decline. Gao *et al.* [25] revealed that the risk of developing dementia was significantly higher in those who have not been diagnosed with diabetes but with glycated haemoglobin equal to or higher than 7% and there is further evidence [26] linking type 2 diabetes and longer duration with cognitive impairment and decline.

Lin and Sheu [27] found that in adults with diabetes who had prior in-hospital diagnosis of hypoglycaemia, the risk of developing dementia was almost three times more, while Feinkohl *et al.* [28] observed that severe hypoglycemia at baseline and during follow-up was associated with higher risk of cognitive decline.

### 3.2. Effect of Dementia and Cognitive Decline on Self Management of Diabetes

Three studies assessed the impact of dementia and cognitive decline on self-management of diabetes. The primary outcomes of these studies [29,30,31] were that, cognitive impairment and dementia were associated with hypoglycaemia, inadequate control of diabetes and worse self-care. One of these studies [29] reported that, cognitive impairment and dementia were independently associated with greater risk of hypoglycaemia.

For example, Feil *et al.* [29] revealed that dementia and cognitive impairment were risk factors for hypoglycaemia particularly in patients who are on insulin. Further study [30] found significant association between cognition and diabetes self management. In addition, Grober *et al.* [31] showed that impairment in memory and executive dysfunction were associated with inadequate control of diabetes.

## 4. Discussion

Evidence from studies reviewed has shown that diabetes is a risk factor in the development of dementia which is often accompanied by a progressive decline in cognitive function. It is expected that patients with diabetes should be involved in the process of taking control and making decisions about aspects of their diabetic care which has been defined as diabetes self management [14]. However, a cognitive impairment may have repercussions for the patient with diabetes with potential for poor self management, leading to further complications including inadequate control of diabetes, hypoglycaemia and hyperglycaemia [27,28]. In addition, the processes and pathways linking diabetes to cognitive impairment and dementia are varied and involve the role of insulin and chronic hyperglycaemia. This association appears bidirectional and may proceed to a vicious cycle where diabetes could lead to dementia and dementia causing further complications of diabetes. This is possible because diabetes care involves self management and a decline in cognitive function in patients with diabetes and dementia may result in poor self management. Therefore, a poorly managed diabetes may exacerbate dementia and cognitive impairment through the possible effect of hyperglycaemia and hypoglycemia. These bidirectional relationships are further discussed below under different subheadings and supported with evidence.

### 4.1. The Association between Diabetes, Cognitive Decline and Dementia

The papers reviewed [8,22,23,24,25,26,27,28] showed the association between diabetes, cognitive impairment and dementia. Epidemiological studies, such as the Rotterdam Study [19] and more recently the Hisayama Study [32] have also established the link between diabetes mellitus and the increased risk of developing dementia. Diabetes is a condition caused by multiple aetiology. While Type 1 diabetes results from autoimmune condition leading to pancreatic beta cell destruction and it is primarily of genetic origin, type 2 diabetes is influenced by both genetic and environmental factors, including poor dietary habits, age, ethnicity and lack of physical activity [1]. In type 2 diabetes, the gradual erosion of beta cell function leads to increased hyperglycaemia while the resistance to the action of insulin could lead to hyperinsulinaemia. A combination of beta cell dysfunction and/or insulin resistance may lead to chronic hyperglycaemia and glucose toxicity which have profound implications for the body, including the brain and therefore, cognition. The toxic effect of high glucose concentration found in patients with diabetes may have effect on the neurons in the brain through osmotic insults and oxidative stress, and continued chronic hyperglycaemia, which leads to the formation of advanced glycation end products (AGE) [33]. AGE coupled with free radicals can cause oxidative damage which can in turn lead to neuronal injury.

In large-scale epidemiological studies, diabetes has been found to be a significant risk factor for age related cognitive impairment, cognitive decline and dementia [26]. The impact of diabetes on the vascular system may also contribute to cognitive impairment and dementia.

The mechanism by which insulin affects memory may relate to a number of pathways. Firstly is the role of insulin in cerebral energy metabolism [34]. It is thought that insulin increases the translocation of GLUT4 (Glucose transporter), which is present in the brain [34]. Therefore, a dysfunction of the beta cell in diabetes can disrupt insulin secretion, reduce the level of insulin in the brain and may affect this mechanism thus leading to glucose dysregulation [33]. In addition, impaired insulin signalling found in diabetes could also affect this process causing a derangement of glucose metabolism which can impact on neuronal development, learning and memory [34].

The effect of type 2 diabetes, dislipidaemia and hyperinsulinaemia may lead to abnormal metabolism of amyloid-β, which is significant in the development of cerebrovascular dysfunction [10]. Furthermore, insulin has direct role in the metabolism of amyloid-β and the effect of abnormal glucose metablosim may lead to the production of AGEs which contribute to the development of diabetes and dementia [10].

Type 2 diabetes also contribute to cerebrovascular dysfunction through ischemia of the microvascular system and endothelial dysfunction leading to chronic cerebral hypoperfusion and these changes may affect regional cerebral blood flow that impairs cerebral protein synthesis, a key factor for learning and memory [10].

According to Umegaki *et al.* [33], the major functions of insulin in the brain are control of food intake and cognitive functioning such as memory; these are affected in insulin resistant states. For example, following insulin resistance and hyperinsulinaemia which are common features of type 2 diabetes, the transport of insulin into the brain across the blood brain barrier is reduced and this lowers the insulin levels in the brain [33]. Therefore, chronic peripheral hyperinsulinaemia may lower brain insulin levels and this could reduce insulin degrading enzyme levels in the brain thereby impairing amyloid—β clearance [34].

There is evidence of a shared pathogenesis between diabetes and Alzheimer’s disease which has been termed as Type 3 diabetes [35,36,37,38]. These links appear to centre around the acetylcholine, amyloid and tau, inflammation, mitochondria and oxidative stress and advanced glycation end products [10,20,34,37].

Insulin regulates the central nervous system levels of acetylcholine and norepinephrine, neurotransmitters which influence cognitive function [34]. It is clear that insulin plays a significant role in neurological function through its effect on choline acetyl transferase, an enzyme responsible for acetylcholine production [37]. Acetylcholine, a neurotransmitter is responsible for cognition and memory formation, thus, a dysfunction in insulin production and insulin resistance could lead to a decrease in acetylcholine levels which may have repercussions for cognition and memory [36].

The accumulation of amyloid beta protein in the brain and pancreatic islet cells is another evidence of the association between diabetes and Alzheimer’s disease and the similarity in their pathogenesis [26,36]. The common understanding of the progression of Alzheimer’s disease is that there is accumulation of neuro-fibrillary plague tangles among the brain neurons [35]. Often, these tangles result from the overproduction of beta-amyloid proteins which the body is unable to remove [35]. These beta-amyloid plagues create neurologic tangles that kill neurons, thus leading to decreased cognition [35].

In addition, the developments of diabetes and Alzheimer’s disease are related to increased oxidative stress and production of advanced glycation end products [36]. AGEs are formed as the end products of Maillard reaction when reducing sugars react non-enzymatically with amino groups of proteins [37]. The metabolic consequences of AGEs include oxidative stress, hypometabolism, impaired cell function, modification of plagues and neurofibrillary tangles, the latter having been implicated in the development of Alzheimer’s disease [36,37].

### 4.2. The Role of Hypoglycaemia in the Development of Dementia

According to Feinkohl *et al.* [28], severe hypoglycaemia was related to significant decline in cognitive function, while Lin and Sheu [27] found that adult participants with diabetes who had previous diagnosis of hypoglycaemia had an almost three-fold increased risk of developing dementia in the subsequent seven-year follow up compared with those without hypoglycaemia. An earlier study by Aung *et al*. [16] found that severe hypoglycaemia was associated with poorer late-life cognitive ability. Although the reasons for the association between hypoglycaemia and cognitive decline remain unclear, a possible explanation is that neuronal cell death may accelerate the process of cognitive decline and dementia by its effect on focal neurological deficits and transient ischaemic attacks [27,28]. In addition, during episodes of hypoglycaemia, higher adrenaline levels increase platelet activation, leucocyte mobilisation, blood coagulability and impairment of endothelial function [27]. In patients with type 1 diabetes, vessel wall stiffness was found to be greater during hypoglycaemia in patients with diabetes of longer duration than those with diabetes of shorter duration.

While the above studies suggest that repeated exposure to severe hypoglycaemia resulted in cognitive impairment in people with diabetes, the prospective Epidemiology of Diabetes Interventions and Complications (EDIC) study showed no association between the frequency of severe hypoglycaemia and cognition over the follow-up period of 18 years [39]. Feinkohl *et al.* [28] provided explanations for the differences observed including the fact that participants in the Diabetes Interventions and Complications Trial were selected because they were highly compliant with treatment and had low risk of hypoglycaemia. Therefore, the results in the EDIC study could have been limited by the low prevalence of hypoglycaemia and age-related cognitive decline.

### 4.3. Impact of Dementia and Cognitive Impairment on Self Management of Diabetes

The approaches to managing diabetes in patients with dementia could be preventative or involve the actual treatment of diabetes. These strategies may include interventions to increase insulin sensitivity, reduction of insulin levels, lifestyle interventions (diet and exercise), use of oral hypoglycaemic agents (metformin, sulfonylureas and thiazolidinediones) and insulin [40].

While peripheral hyperinsulinaemia contribute to the development of Alzheimer’s disease, the administration of intranasal insulin, which travels directly into the central nervous system, may enhance cognition in patients with early Alzheimer’s disease or Mild Cognitive Impairment [10]. Impairment in cognitive ability may affect the capacity of the patients with diabetes to take effective control of their condition and engage in the management strategies outlined above. The studies relating to the impact of dementia and cognitive decline on self-management of diabetes showed that cognitive decline and dementia were linked to hypoglycaemia, inadequate control of diabetes and worse self-care [16,27,28]. Conversely, hypoglycaemia has been shown to be associated with increased risk of cognitive impairment, further strengthening the bidirectional relationship between diabetes, cognitive decline and dementia [27,28].

According to Acee [9], diabetes self-management refers to the patient’s responsibility and involvement in almost all aspects of his or her diabetes care and management. However, patients with diabetes and declining cognitive function may present with difficulties in managing self-care [3], including failure to effectively administer their medications such as oral tablets and insulin, monitoring, adherence to diet and exercise regimes. These can lead to the development of diabetic complications including hypoglycaemia, hyperglycaemia, diabetic ketoacidosis and hyper osmolar non ketotic state. Impairment in language, confusion and disorientation are common symptoms of dementia and hypoglycaemia and these similarities in symptoms can affect diagnosis and management of these conditions [3]. Similarly, discomfort or pain has been shown to be difficult to express in people with dementia and this is not uncommon in diabetic patients, especially those with diabetic neuropathy. According to Sanz *et al*. [8], in patients with Alzheimer’s disease and diabetes, difficulties in performing activities of daily living which are known to cause functional decline are part of the symptoms found in these patients.

However, empowerment programmes that rely on the use of structured education approaches such as Diabetes Education and Self Management for Ongoing and Newly Diagnosed (DESMOND), X-pert and Dose Adjustment for Normal Eating (DAFNE) that incorporate lifestyle changes in the management of diabetes could help ameliorate some of these challenges [41,42,43].

## 5. Conclusions

The prevalence of diabetes and dementia is on the increase in the UK and worldwide partly due to the increase in the ageing population and lifestyle choices. In addition, there is evidence of the association between diabetes, dementia and cognitive decline including the shared pathogenesis between diabetes and Alzheimer’s disease.

This review has also shown that diabetes is a risk factor for dementia and cognitive decline. Conversely, cognitive impairment and dementia have a significant impact on self-management of diabetes. In particular, hyperglycaemia and hypoglycaemia may result from poor self management of diabetes due to a decline in cognitive function further increasing the risk of diabetic complications. The association between diabetes, cognitive decline and dementia appears bidirectional and this could lead to a vicious cycle. Further studies are required to fully establish the link between diabetes, dementia and cognitive decline.

### Relevance to Clinical Practice

Patients with diabetes who have dementia may benefit from structured education programmes such as DESMOND and DAFNE. Lifestyle approaches have been found to be effective in reducing the risk of developing type 2 diabetes and this is essential in the prevention and management of dementia due to the lack of effective treatment options for this condition [1,9]. This should reduce a deterioration in cognitive decline and enhance self-management of diabetes. In addition, the recognition of cognitive decline should bring into focus the need for education strategies.

Peripheral insulin administration as the only strategy for managing diabetic patients with Alzheimer’s disease may increase the risk of hypoglycaemia and hyperinsulinaemia in these patients. Therefore, the use of intranasal insulin which is absorbed directly to the central nervous system provides additional option. Due to the possible link between hypoglycaemia and dementia, it is useful to balance the need for treatment with insulin and sulphonylureas and the risk of developing hypoglycaemia in patients who have diabetes and dementia.
